# Genetic noise control via protein oligomerization

**DOI:** 10.1186/1752-0509-2-94

**Published:** 2008-11-03

**Authors:** Cheol-Min Ghim, Eivind Almaas

**Affiliations:** 1 Microbial Systems Biology Group, Biosciences and Biotechnology Division, Lawrence Livermore National Laboratory, 7000 East Avenue Livermore, CA 94550, USA

## Abstract

**Background:**

Gene expression in a cell entails random reaction events occurring over disparate time scales. Thus, molecular noise that often results in phenotypic and population-dynamic consequences sets a fundamental limit to biochemical signaling. While there have been numerous studies correlating the architecture of cellular reaction networks with noise tolerance, only a limited effort has been made to understand the dynamic role of protein-protein interactions.

**Results:**

We have developed a fully stochastic model for the positive feedback control of a single gene, as well as a pair of genes (toggle switch), integrating quantitative results from previous *in vivo *and *in vitro *studies. In particular, we explicitly account for the fast binding-unbinding kinetics among proteins, RNA polymerases, and the promoter/operator sequences of DNA. We find that the overall noise-level is reduced and the frequency content of the noise is dramatically shifted to the physiologically irrelevant high-frequency regime in the presence of protein dimerization. This is independent of the choice of monomer or dimer as transcription factor and persists throughout the multiple model topologies considered. For the toggle switch, we additionally find that the presence of a protein dimer, either homodimer or heterodimer, may significantly reduce its random switching rate. Hence, the dimer promotes the robust function of bistable switches by preventing the uninduced (induced) state from randomly being induced (uninduced).

**Conclusion:**

The specific binding between regulatory proteins provides a buffer that may prevent the propagation of fluctuations in genetic activity. The capacity of the buffer is a non-monotonic function of association-dissociation rates. Since the protein oligomerization *per se *does not require extra protein components to be expressed, it provides a basis for the rapid control of intrinsic or extrinsic noise. The stabilization of regulatory circuits and epigenetic memory in general is of direct implications to organism fitness. Our results also suggest possible avenues for the design of synthetic gene circuits with tunable robustness for a wide range of engineering purposes.

## Background

Recent experiments on isogenic populations of microbes with single-cell resolution [1-3] have demonstrated that stochastic fluctuations, or noise, can override genetic and environmental determinism. In fact, the presence of noise may significantly affect the fitness of an organism [4]. The traditional approach for modeling the process of molecular synthesis and degradation inside a cell is by deterministic rate equations, where the continuous change of arbitrarily small fractions of molecules is controlled instantaneously and frequently represented through sigmoidal dose-response relations. However, the rate-equation approaches can not explain the observed phenotypic variability in an isogenic population in stable environments. In particular, when molecules involved in feedback control exist in low copy numbers, noise may give rise to significant cell-to-cell variation as many regulatory events are triggered by molecules with very low copy numbers ≲ 100 [5]. A well known example is the regulation of inorganic trace elements [6], such as iron, copper, and zinc. While these trace elements are essential for the activity of multiple enzymes, their presence may quickly turn cytotoxic unless their concentrations are carefully controlled.

Although the presence of phenotypic variation due to stochastic fluctuations need not be detrimental for a population of cells [7], elaborate regulatory mechanisms have evolved to attenuate noise [8]. Several systems-biology studies have recently focused on a select set gene-regulatory circuits, in particular those with feedback control. Feedback control circuits have been identified as important for multiple species and proven responsible for noise reduction and increased functional stability in many housekeeping genes through negative autoregulation [9], long cascades of ultrasensitive signaling [10], bacterial chemotaxis [11], and the circadian clock [12]. Additionally, recent studies on iron homeostasis [13,14] in *E. coli *highlight the noise-reducing capability mediated by small RNAs.

Here, we study reversible protein-protein binding as a novel source for genetic noise control. In particular, we have quantitatively analyzed the effects of protein oligomerization on noise in positive autoregulatory circuits as well as a simple toggle-switch [15]. The all-or-none threshold behavior of positive-feedback circuits typically improves robustness against "leaky" switching. However, due to their functional purposes, gene circuits involved in developmental processes or stress responses that often accompany genome-wide changes in gene expression are intrinsically noisier than negative feedback circuits.

It is frequently observed that transcription factors exist in oligomeric form [16], and protein oligomerization is an important subset of protein-protein interactions, constituting a recurring theme in enzymatic proteins as well as regulatory proteins. Well studied examples include the *λ*-phage repressor, *λ*CI (dimer), the TrpR (dimer), LacR (tetramer), and Lrp (hexadecamer or octamer). While many of the RNA-binding proteins dimerize exclusively in the cytosol, the LexA repressor [17], the leucine-zipper activator [18,19], and the Arc repressor [20] have been shown to form an oligomer either in the cytosol ("dimer path") or on the DNA by sequential binding ("monomer path"). Previously, the efficacy of monomer and dimer transcription-regulation paths to reduce noise was separately studied for a negative-feedback autoregulatory circuit [21]. In contrast, we have focused on oligomerization in positive-feedback autoregulatory circuits, as well as genetic toggle switches based on the mutual repression of genes [15]. We find that cytosolic transcription-factor oligomerization acts as a significant buffer for abundance-fluctuations in the monomer, overall reducing noise in the circuit. Additionally, the noise-power spectral density is shifted from the low-to the high-frequency regime. In the toggle switch, cytosolic oligomerization may significantly stabilize the functional state of the circuit. This is especially evident for heterodimerization.

Yet another interesting case of ligand-binding-mediated receptor oligomerization has been reported [22,23], where the formation of various structures of oligomers may act to buffer the intracellular signaling against noise. Although our modeling and analysis is based on prokaryotic cells, we expect our main findings to be organism independent since protein oligomers, especially homodimers, is such a common occurrence across the species [24], with homodimers comprising 12.6% of the high-fidelity human proteome [25,26].

## Results and Discussion

### Dimerization breaks long-time noise correlations in autogenous circuit

**Figure 1:**
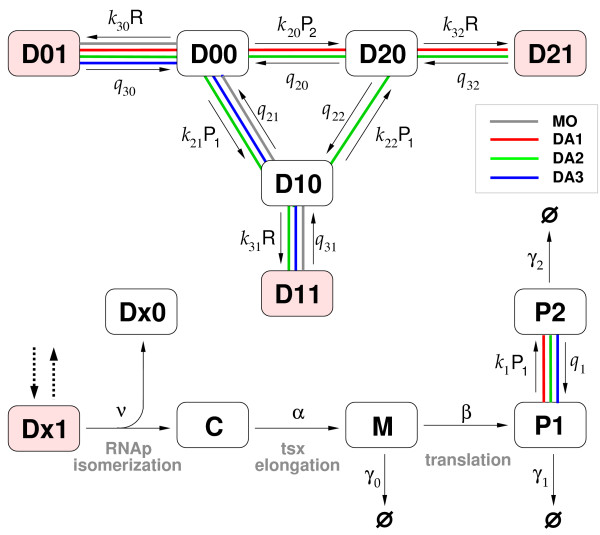
**Schematic of model autoregulation gene circuit**. The DNA binding status is indicated by Dxy, where x corresponds to the operator region (empty = 0, monomer = 1, dimer = 2), and y to the promoter region (empty = 0, RNA polymerase bound = 1). C represents the open complex of DNA-RNAp holoenzyme with the promoter sequence just cleared of RNAp and is subject to transcription elongation. Finally, M, P1 and P2 correspond to mRNA, protein monomer, and dimer, respectively. The network topologies can be grouped into two classes, monomer-only (MO) or dimer-allowed (DA) circuits. We have studied DA1 (red lines), which only allows the dimer to bind with the DNA-operator sequence, DA2 (green) with sequential binding of monomers on the DNA, and DA3 (blue), which shares protein-DNA binding kinetics with MO while allowing dimerization in the cytosol. Note that for topology DA2, we have chosen *K*_31 _= *K*_30 _(see text for details) We have assumed cells to be in the exponential growth phase and the number of RNAp (R) constant.

To evaluate the dynamic effects of protein-protein binding in positive-autoregulation gene circuits, we construct several alternative models of positive autogenous circuits. Each model emphasizes a different combination of possible feedback mechanisms, and the network topologies considered can be grouped into the two classes of monomer-only (MO) and dimer-allowed (DA) circuits, according to the availability of a protein-dimer state (color coding in Fig. 1). We further group the DA circuits into three variations, DA1 through DA3, depending on which form of the protein is the functional transcription factor (TF) and where the dimerization occurs. For DA1, we only allow the dimer to bind with the DNA-operator sequence (dimeric transcription factor, DTF), while for DA2 dimerization occurs through sequential binding of monomers on the DNA. In DA3, the protein-DNA binding kinetics is the same as in the MO circuit, hence monomeric transcription factor (MTF), with the addition of a cytosolic protein dimer state. While we will only present results for DA1 in this paper, there is no significant difference for DA2 and DA3 [Additional file 1].

Note that the feedback loop is not explicit in Fig. 1 but implicitly included through the dependence of RNAp-promoter binding equilibrium on the binding status of the TF-operator pair. The sign (positive or negative) and strength of the feedback control is determined by the relative magnitude of the dissociation constants between RNAp and DNA which is either free or TF-bound. For instance, topology DA1 has positive feedback control if *K*_30 _= *k*_30_*/q*_30 _> *K*_32 _= *k*_32_*/q*_32_, and *K*_30 _corresponds to the level of constitutive transcription (transcription initiation in the absence of bound transcription factor). For each topology, we study the dependence of noise characteristics on the kinetic rates by varying the dimer lifetime, binding affinity, and the individual association/dissociation rates (see Table and Fig. 1). While we only discuss positive feedback control of the autogenous circuit in this paper, we have obtained corresponding results for negative feedback control [Additional file 1].

**Figure 2:**
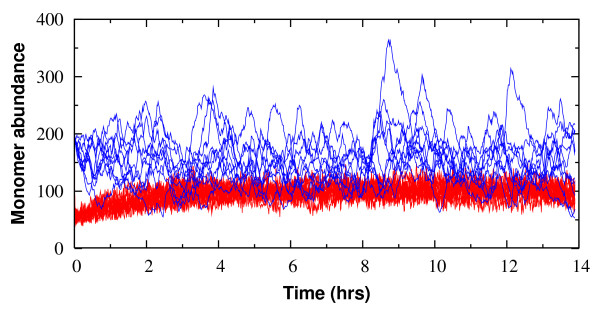
**Ten independent time courses of the abundance of protein monomers in the (positive) autoregulatory circuit**. The availability of a cytosolic dimer state (red, using circuit topology DA1) significantly reduces the copy-number fluctuations of the monomer compared to the monomer-only (MO) circuit (blue). All corresponding MO and DA1 parameters have the same values. In the ensuing simulations initial conditions are chosen to be the steady state solution of the corresponding deterministic rate equation so that the transient behavior should be minimized.

Fig. 2 shows a sample of ten representative time courses for the protein abundance. The effect of stochastic fluctuations is marked in the MO circuit. However, in all the DA circuits where the protein may form a cytosolic dimer we observe a significantly reduced level of noise in the monomer abundance. The suppression of fluctuations persists throughout the range of kinetic parameters that (so far) is known to be physiologically relevant (see Table 1).

**Table 1:** Probability rates for positive autogenous circuit

Category	Symbol	Reaction	Value (s^-1^)	Ref.
protein dimerization	*k*_1_	P_1 _+ P_1 _→ P_2_	0.001–0.1	[51,52]
	*q*_1_	P_2 _→ P_1 _+ P_1_	0.1–1	

TF-operator int	*k*_20_	P_2 _+ D00 → D20	0.012	[53-55]
	*q*_20_	D20 → P_2 _+ D00	0.9	
	*k*_21_	P_1 _+ D00 → D10	0.038	
	*q*_21_	D10 → P_1 _+ D00	0.3	
	*k*_22_	P_1 _+ D10 → D20	0.011	
	*q*_22_	D20 → P_1 _+ D10	0.9	

RNAp-promoter int	*k*_30_	R + D00 → D01	0.038	[56-58]
	*q*_30_	D01 → R + D00	0.3	
	*k*_31_	R + D10 → D11	0.038^†^, 0.38^‡^	
	*q*_31_	D11 → R + D10	0.3^†^, 0.03^‡^	
	*k*_32_	R + D20 → D21	0.38^*†^	
	*q*_32_	D21 → R + D20	0.03^*†^	

Isomerization	*ν*	Dx1 → C + Dx0	0.0078	[55]

tsx-tsl elongation & decay	*α*	C → M + R	0.03	[59,60]
	*β*	M → P_1 _+ M	0.044	
	*γ*_0_	M → ∅	0.0039	
	*γ*_1_	P_1 _→ ∅	7 × 10^-4^	
	*γ*_2_	P_2 _→ ∅	0.7–3.5 × 10^-4^	

Kinetic rates for the positive autogenous circuit. Experimentally available rates are all taken from lambda phage-*E. coli *complex. The values with superscript correspond to the circuit topologies DA1 (*), DA2 (†), and DA3 (‡) in Fig. 1.

**Figure 3:**
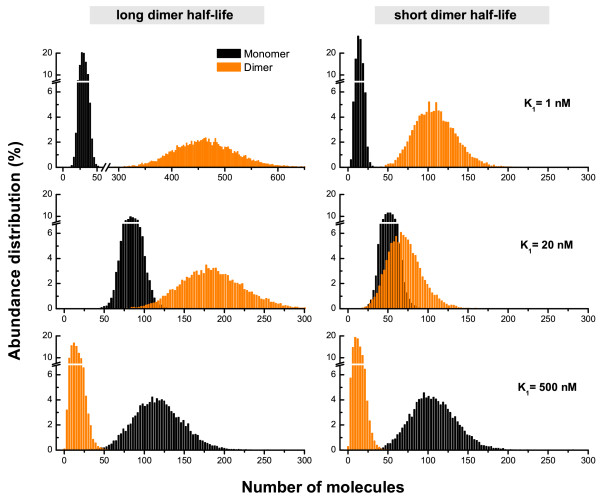
**Stationary state distribution of monomer (black) and dimer (orange) protein abundance in the positive autogenous circuits**. The left (right) column corresponds to a ratio of the dimer and monomer decay rates of *γ*_2_/*γ*_1 _= 1/10 (*γ*_2_/*γ*_1 _= 1/2). The molecular copy numbers are collected at a fixed time interval (5·10^3 ^sec) after the steady state has been reached. Here *K*_1 _≡ *q*_1_/*k*_1 _is the dissociation constant of the protein dimer. As the binding equilibrium is shifted towards the dimer state (decreasing *K*_1_), the noise level is monotonically reduced (see Table 2). Note that the prolonged protein lifetime due to the complex formation (left column) affects the noise level.

Calculating the steady-state distribution for the monomer and dimer abundances (Fig. 3) we observe a clear trend that the monomer Fano factor (variance-to-mean ratio) is reduced as the binding equilibrium is shifted towards the dimer. This trend is conserved for all the investigated DA topologies (see *Supplementary Information*). As long as dimerization is allowed in the cytosol, the fast-binding equilibrium absorbs long-time fluctuations stemming from bursty synthesis or decay of the monomer. When a random fluctuation brings about a sudden change in the monomer copy number, dimerization provides a buffering pool that absorbs the sudden change. Otherwise, random bursts in the monomer abundance will propagate to the transcriptional activity of the promoter, leading to erratic control of protein expression. It should be emphasized that this has nothing to do with the sign of regulation and is in agreement with the observations of Ref. [21] for negative autoregulation. Surprisingly, the magnitude of noise reduction in the positive autoregulatory circuit is nearly the same as that for negative autoregulation which is typically considered a highly stable construct [Additional file 1].

A heuristic explanation can be found from Jacobian analysis of a deterministic dynamical system, which is justified for small perturbations around a steady state. When a random fluctuation shifts the monomer copy number away from its steady-state value, the decay toward the steady state can be described by the system Jacobian. The disparity in the magnitude of the (negative) eigenvalues of the Jacobian matrix for the MO versus the DA circuits signifies that the perturbed state is buffered by fast settlement of the monomer-dimer equilibrium. This buffering occurs before random fluctuation can accumulate, possibly with catastrophic physiological effects, explaining the coarse long-time patterns observed in the MO model in contrast with the DA circuits (Fig. 2).

### Frequency-selective whitening of Brownian noise

**Figure 4:**
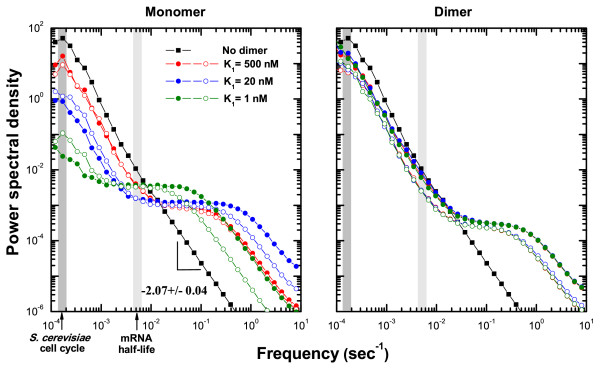
**Power spectral density (PSD) of fluctuations in protein abundance**. The PSD of the MO circuit clearly displays a power-law behavior. All other model systems with an available cytosolic protein dimer state (DA1 shown here) develop a plateau in the mid-frequency region regardless of the model details (see *Supplementary Information*). As the dimer binding affinity increases, the noise level is further reduced. We have included the MO result in the dimer panel (right) for reference. Datasets with solid (empty) symbols correspond to *γ*_2_/*γ*_1 _= 1/10 (*γ*_2_/*γ*_1 _= 1/2).

The dimerization process itself generates stochastic fluctuations on a short time scale. However, since this time scale is essentially separated from that of monomer synthesis and decay (orders of magnitude faster), dimerization effectively mitigates monomer-level fluctuations. The frequency content of the fluctuations is best studied by an analysis of the power spectral density (PSD), which is defined as the Fourier transform of the autocorrelation function [27], originally introduced for signal processing. Fig. 4 shows the noise power spectra of DA1, and the distinction between the MO circuit and the DA topology is immediately evident. In particular, we note the following two features. (i) A power-law decay with increasing frequency and (ii) a horizontal plateau for the DA circuits. The power-law feature is explained by the "random walk" nature of protein synthesis and decay: The power-law exponent is approximately 2, which is reminiscent of Brownian motion (a Wiener process) in the limit of large molecular copy numbers. Compared to other commonly observed signals, such as white (uncorrelated) noise or 1/*f *noise, protein synthesis/decay has a longer correlation time. If the autocorrelation function of a time course is characterized by a single exponential decay, as is the case for Brownian noise, the PSD is given by a Lorentzian profile, and thus, well approximated by an inverse-square law in the low-frequency regime. We do not observe a saturation value for the MO circuit, and it is likely not in the frequency window of physiological interest. This may especially be the case for circuits where the correlation times are long.

The noise reduction is in the physiologically relevant low-frequency regime, and in Fig. 4 we have indicated the typical values for a cell cycle and mRNA lifetime. Although stochastic fluctuations impose a fundamental limit in cellular information processing, multiple noise sources may affect cellular physiology non-additively. For a living cell, fluctuations are especially relevant when their correlation time is comparable to, or longer than, the cell cycle. At the same time, short-time scale fluctuations (relative to the cell cycle) are more easily attenuated or do not propagate [28]. Additionally, the observed flat region in the PSD of the DA circuits implies that as far as mid-range frequency fluctuations are concerned, we can safely approximate them as a white noise. This insight may shed light on the reliability of approximation schemes for effective stochastic dynamics in protein-only models.

### Increased lifetime of dimer plays an important role

The virtue of the cytosolic dimer state is also directly related to the extended lifetime of proteins when in a complex. Except for the degradation tagging for active proteolysis, a much slower turnover of protein oligomers is the norm. This is partly explained by the common observation that monomers have largely unfolded structures, which are prone to be target of proteolysis [29]. It has also been pointed out that the prolonged lifetime of the oligomeric form is a critical factor for enhancing the feasible parameter ranges of gene circuits [30]. As seen from Fig. 3 (also Table 2), the fold change of the noise reduction, while still significant, is not as strong for the (hypothetical) case of dimer lifetime being the same as that of the monomer (*γ*_2_/*γ*_1 _= 1/2). However, the low-frequency power spectra still exhibit almost an order-of-magnitude smaller noise power than in the MO circuit with the same rate parameters (Fig. 4). Hence, the noise reduction capability holds good as long as the dimer lifetime is kept sufficiently long compared with the monomer-dimer transition.

**Table 2:** Relative Fano factors of protein abundance distributions

	*γ*_2 _= *γ*_1_/10		*γ*_2 _= *γ*_1_/2	
	
*K*_1 _(nM)	monomer	dimer	monomer	dimer
1	0.127	0.809	0.132	0.679
20	0.209	0.936	0.230	0.716
500	0.866	0.478	0.826	0.426

The Fano factor of protein abundance distribution for the autogenous circuits (topology DA1), relative to that of the monomer-only (MO) circuit, 8.729.

### Effects of homo-dimerization in genetic toggle switch

The exceptionally stable lysogeny of the phage *λ*, for which the spontaneous loss rate is ≲ 10^-7 ^per cell per generation [31,32], has motivated the synthesis of a genetic toggle switch [15]. Toggle switch is constructed from a pair of genes, which we will denote as gene *A *and *B*, that transcriptionally repress each other's expression. This mutual negative regulation can be considered an effective positive feedback loop and provides the basis for the multiple steady states. The existence of multistability, in turn, may be exploited as a device for epigenetic memory or for decision making [33].

As the general attributes of positive feedback with cooperativity suggest, a genetic toggle switch responds to external cues in an ultrasensitive way: When the strength of a signal approaches a threshold value, the gene expression state can be flipped by a small change in the signal. For example, the concentration of protein *A *(*B*) may rapidly switch from high to low and vice versa. However, previous studies of a synthetic toggle switch have shown that the noise-induced state switching is a rare event [15,34,35]. In the ensuing analysis, we aim to delineate the origin of this exceptional stability.

In a simple model, the monomer-only (MO) toggle, regulatory proteins only exist in monomeric form. Although an external signal is not explicitly included, random fluctuations in the abundance of the circuit's molecular components will occasionally flip the toggle-state for the two protein species. Drawing on the results from our analysis of positive autoregulatory gene circuits, we hypothesize that dimerization in the regulatory proteins of the toggle switch will serve to stabilize its performance against noise. We allow the protein products of each gene to form a homodimer, being either *AA *or *BB*, which is similar to the cI-cro system in phage *λ *[36]. The dissociation constant for the dimers is defined as *K*_1 _= *q*_1_/*k*_1_, where *k*_1 _is the rate of two monomers forming a complex, and *q*_1 _the rate of the complex breaking up into its two constituents.

**Figure 5:**
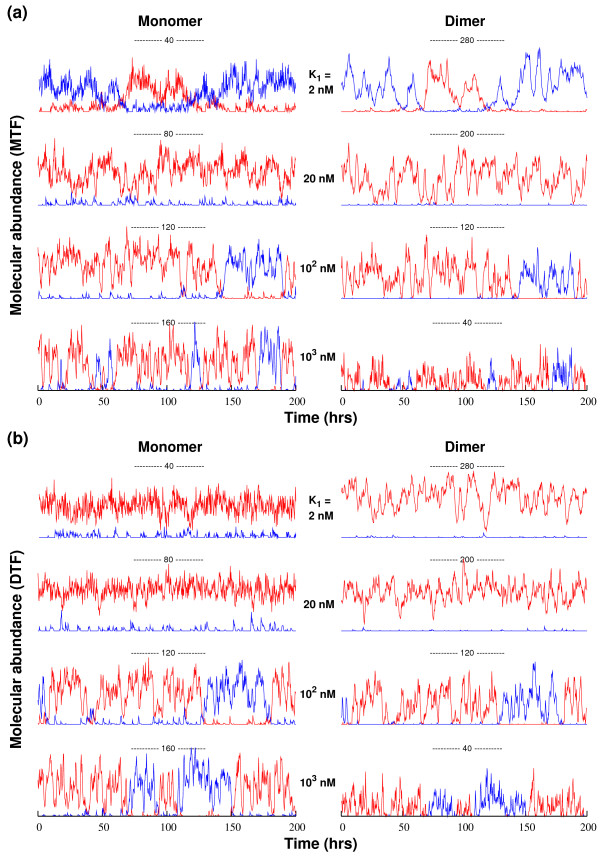
**Sample time series of monomer and dimer copy numbers in genetic toggle switch**. (a) MTF circuit, where monomer is the functional form of the repressor. (b) DTF circuit, where dimer is the functional form of the repressor. The left (right) column shows the number of the two monomer molecules *A *and *B *(dimers *AA *and *BB*), and the initial state is always with species *A *(red) in high abundance. Note that the switching frequency depends on the binding affinity of protein dimer.

We evaluate the effect of the fast protein binding-unbinding dynamics on the toggle switch performance by using either (i) the monomers or (ii) the homodimers as the functional form of the repressor. Fig. 5 shows, for selected values of the dissociation constant *K*_1_, representative time series of the protein monomer (left) and dimer (right) abundances for the case of (a) monomeric or (b) dimeric transcription factors, respectively. A careful analysis of the phase space (in presence of noise) for our chosen set of parameters confirms that the studied toggle-switch systems are in the bistable region [37].

When monomer is the functional form of the repressor molecule (Fig. 5(a)) and *K*_1 _is large (limit of low dimer affinity), the protein populations are dominated by monomers. Hence, the circuit effectively behaves as an MO toggle. As *K*_1 _decreases, we see that the level of random switching is suppressed: Analogous to the autogenous circuit, the dimer pool stabilizes the protein monomer population. However, the noise suppression is not monotonic with increasing dimer binding affinity. Indeed, for very large binding affinities (small *K*_1_), the number of random switching events is increased since the monomer is only available in low copy numbers. Consequently in this limit, it becomes more likely that a small fluctuation in the monomer abundance can cause a dramatic change in the overall gene expression profile. The noise-stabilizing effect of dimerization is also reflected in the corresponding PSDs [Additional file 1]. For instance, we observe a marked suppression of low-frequency fluctuations in the monomer abundance with increasing *K*_1_.

In Fig. 5(b) we show corresponding sample time series for the case of a dimeric repressor, all other properties being the same as in (a). While the overall trends are similar, we do note the following difference. Contrary to the monomeric repressor case, there are very few toggle events in the strong binding limit: Since the signaling molecules (dimers) of the dominant gene (the "on"-gene) tend to exist in large copy numbers, a significant fluctuation is needed to flip the state of the toggle switch. In the case of monomeric repression, the signaling molecule exists in low abundance in this limit. Thus, the dominant protein species in the dimeric-repressor system is able to maintain much better control over the state of the toggle switch.

**Figure 6:**
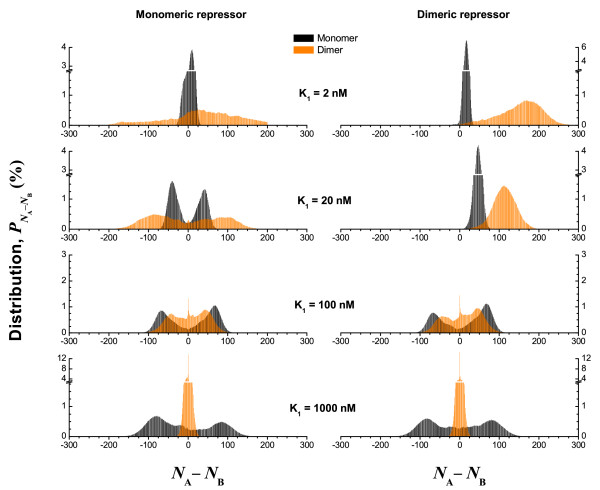
**Distribution of monomer abundance differences between protein species *A *and *B***. The asymmetry with respect to the zero axis is due to the choice of initial state (species *A *high) and the finite time span of simulations.

In Fig. 6, we show the distribution (*N*_*A *_- *N*_*B*_), the difference in molecule abundance for the two protein species in the case of monomeric (left) and dimeric (right) transcription factor. The asymmetry with respect to the zero axis is caused by our choice of initial conditions (protein species *A *in high concentration and species *B *in low concentration), as well as the finite length of the time series. For monomeric transcription, the presence of dimers with moderate binding affinity sharpens the monomer abundance distribution while accentuating its bimodal character. This is in agreement with the qualitative observation from Fig. 5 on switching stability. For dimeric transcription, we clearly observe that the symmetry of the system is broken for small values of *K*_1_, indicating that the state of the toggle switch is extremely stable, and hence, likely determined by the choice of the initial conditions.

**Figure 7:**
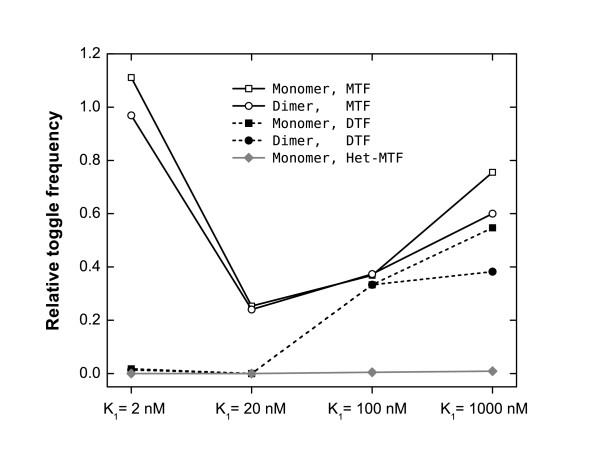
**Random switching rates of genetic toggle switches**. Ordinate is the ratio of the random switching rates of various toggle switches to that of the monomer-only (MO) circuit, 7.5 × 10^-6^/hour. MTF, monomeric transcription factor; DTF, dimeric transcription factor; Het-MTF, monomeric transcription factor with deactivated heterodimer state.

To systematically quantify our observations on the interplay between dimer-binding affinity and the functional stability of the toggle switch, we generated long time series (≈3·10^7 ^sec) to measure the average spontaneous switching rate. In Fig. 7, we show the average toggle frequency relative to that of the MO toggle for the binding affinities *K*_1_/nM = {2, 20, 100, 1000}, and the average MO switching rate is 7.5 × 10^-6^/hour. As expected, we find that intermediate values of *K*_1 _are able to stabilize the toggle switch. Fig. 7 also highlights the increased stability of the toggle switch for a dimeric versus monomeric transcription factor, the dimeric switching rates always being lower and approaching zero for strong dimer binding.

### Heterodimerization in genetic toggle switch

We have also considered the case of heterodimerization in the toggle switch, since the noise- and functional stabilization of the switch may be directly affected by the composition and source of the dimers. Note that, the gene-regulation activity is conferred by the two monomer proteins *A *and *B *and not the heterodimer *AB*. However, we find that the presence of (inactive) heterodimers gives rise to very similar noise-stabilizing effects as that of homodimers (Fig. 7). In fact, the existence of heterodimer state allows the dominant protein species to effectively suppress the (active) monomers of the minority species. Thus the heterodimer circuit shows dramatically enhanced functional stability as compared to the case of homodimeric repressors, not sharing the discussed vulnerability of MO circuit to intrinsic noise. Although, to our knowledge, this is a purely hypothetical toggle-switch design, it provides a general strategy for noise control in synthetic gene circuits, along with previously proposed approach of overlapping upstream regulatory domains [38].

## Conclusion

Cells have evolved distinct strategies to combat the fundamental limits imposed by intrinsic and environmental fluctuations. We investigated the role of protein oligomerization on noise originating from the random occurrence of reaction events and the discrete nature of molecules. Recent efforts to correlate network structure with functional aspects may provide valuable insights into approaches for network-level noise control [39]. While negative feedback is one of the most abundantly observed patterns to achieve the goal of stability, it begs the question of how cells reliably change the expression of genes from one state to another. The ultrasensitive response circuit, exemplified by the ubiquitous signal transduction cascades in eukaryotic cells, has been proposed as an answer to this question [40,41].

In addition to the combinatorial expansion of functional specificity, we argue that the availability of oligomeric states contributes to the attenuation of stochastic fluctuations in protein abundance. In positive autoregulatory gene circuits, where the abundance of an expressed protein controls its own synthesis rate, dimerization provides a buffer serving to mitigate random fluctuations associated with the bursty transcription-translation process. We find that short-time binding-unbinding dynamics reduce the overall noise level by converting potentially pathological low-frequency noise to physiologically unimportant, and easily attenuated, high-frequency noise [28].

Noise-induced switching generally signals a defect in cellular information processing. Untimely exit from latency in the lambda-phage system directly implies, as the immediate consequence to viruses, increased chance of being targeted by a host immune system. In the case of a bacterium, the expression of a specific set of sugar uptake genes when the sugar is absent from the external medium is a considerable waste of cellular resources. For example, *lac *operon of *E. coli *can be considered to have the circuitry of mutual antagonism between the lacI gene and lactose uptake-catabolic genes [42]. A difference lies in the non-transcriptional deactivation of the allosteric transcription factor LacI. LacY, lactose permease, indirectly regulates LacI by increasing lactose uptake, which in turn catalytically deactivates LacI. Likewise, many pili operons of Gram-negative bacteria are also known to utilize heritable expression states, which are of crucial role in pathogenesis [43,44].

We expect that the random flipping of gene expression states in the examples of positive-feedback-based genetic switches may very well be closely coupled with the fitness of an organism. Phenomenological models relating the fitness of an organism to random phenotypic switching in fluctuating environments have provided important insights into the role of noise [45], but still many questions remain unanswered. Applying these insights to the design of a synthetic gene switch demonstrates the potential use of affinity-manipulation for synthetic biology, where the construction of genetic circuits with tunable noise-resistance is of central importance. In particular, our analysis highlights the potential utility of heterodimerization to stabilize ultrasensitive switches against random fluctuations. In practice, small ligand molecules may be employed to regulate and tune the binding affinity of regulatory proteins, being either monomers or dimers. Our results further suggest that the structure of the protein-interaction network [33] may provide important insights on methods for genome-level noise control in synthetic and natural systems.

## Methods

### Model construction

To evaluate the general role of protein oligomerization in a broad functional context, we studied the two most common motifs found in genetic regulatory circuits: positive autoregulation and the bistable switch. The reaction scheme studied is summarized in Fig. 1, where the binding/unbinding reactions between RNAp and promoter or between TF and operator are made explicit. Each distinct binding status of DNA is associated with a unique transcription initiation rate, and then the overall rate of mRNA synthesis is a weighted average of the initiation rates for distinct binding status, where the weights are given by the relative abundance of each configuration at equilibrium, determined by the calculation of binding energy [46]. Note that, neither binding equilibrium nor empirical Hill-type cooperativity is assumed *ad hoc*. In particular, we split the lumped transcription process into two separate events, (i) isomerization of closed RNAp-promoter complex to its open form and (ii) transcription elongation followed by termination. This is to reflect the availability of the free promoter while the transcription machinery proceeds along the coding sequence of a gene as soon as the promoter region is cleared of the RNAp holoenzyme. Otherwise, the promoter would be inaccessible during a whole transcription event, altering the random mRNA synthesis dynamics.

To realize the genetic toggle switch in a stochastic setting, we keep track of the microscopic origin of cooperativity that gives rise to bistability. Among various strategies, we employ multiple operator sites which have the same binding affinity with the repressor. The resultant circuitry is, in essence, two autogenous circuits, *A *and *B*, which are connected through the active form of their expressed proteins (the active form being either monomer or dimer). The connection is implemented by allowing the active form of proteins *A *(*B*) to bind the operator sites of gene *B *(*A*). In order to make the interaction between the two genes repressive, unlike positive autogenous circuits, *K*_31 _and *K*_32 _in Fig. 1 are now greater than *K*_30_, making the protein transcriptional repressors. For reasons of analytical simplicity, we have studied the symmetric toggle switch, where the reaction descriptions of each component follow those of the autogenous circuit. Again, the quantitative characteristics of macromolecular binding-unbinding are chosen based on the phage lambda-*E. coli *system. The only exception is related to the multiple operator sites, where the second repressor binds an operator site with higher binding affinity when the first site is already occupied by the repressor protein [47]. We introduce three different dimerization schemes. Three different dimerization scheme have been introduced: (i) homodimerization with monomeric repressor, (ii) homodimerization with dimeric repressor, and (iii) heterodimerization with monomeric repressor. By solving for the stationary states of the deterministic rate equations, we could identify the bistability region in parameter space to which all the model systems under consideration belong.

### Stochastic simulation

While the deterministic rate equation approach or Langevin dynamics explicitly gives the time-evolution of molecular concentration in the form of ordinary differential equations, chemical master equation (CME) describes the evolution of a molecular number state as a continuous-time jump Markov process. To generate the statistically correct trajectories dictated by CME, we used the Gillespie direct [48] and Next Reaction (Gibson-Bruck) [49] algorithms, both based on the exact chemical master equation. The Dizzy package [50] were used as the core engine of the simulations. To ensure that calculations were undertaken in a steady state, we solved the deterministic set of equations for steady state using every combination of parameters investigated. We employed these deterministic steady-state solutions as initial conditions for the stochastic simulations. For each model system, we generated 10^5 ^ensemble runs with identical initial conditions and used the instantaneous protein copy number at a fixed time point *t *= 5000 sec. To achieve high-quality power spectra in the low- and high-frequency limits, we ran time courses (~10^5 ^sec) with higher sampling frequency (20 measure points per sec).

To calculate the average switching rate, we generated time series of minimum length 3·10^7 ^sec (approximately corresponding to 1 year). We identify a state change in the toggle switch by monitoring the ratio of the monomer and dimer abundance for the two protein species. In order to avoid counting short-time fluctuations that do not correspond to a prolonged change of the toggle state, we a applied sliding-window average to the time series, using a window size of 1000 sec.

## Authors' contributions

CMG and EA designed the study. CMG performed the computations. CMG and EA analyzed the results and wrote the paper. Both authors have read and approved the final version of the paper.

## Supplementary Material

Additional file 1 **Supplementary results for the positive autoregulatory circuits with various topology**. Protein abundance distribution and power spectral density of autogenous DA2 and DA3 circuits are presented. Click here for file
