# Supplementary material for: Genetic noise control via protein oligomerization
Source: BMC Syst Biol. 2008 Nov 3;2:94. doi: 10.1186/1752-0509-2-94 (PMC2584638; doi:10.1186/1752-0509-2-94)
Supplement: Additional file 1 — Supplementary results for the positive autoregulatory circuits with various topology. Protein abundance distribution and power spectral density of autogenous DA2 and DA3 circuits are presented. [file 1752-0509-2-94-S1.pdf]

Supplementary Information for

Genetic noise control via protein oligomerization

Cheol-Min Ghim & Eivind Almaas
Biosciences and Biotechnology Division, Lawrence Livermore National Laboratory

1. Results for DA2 and DA3 circuits

We explored the effects of protein homo-dimerization in various topologies of autogenous circuits. Monomer-only (MO) and a dimer-allowed circuit (DA1) are presented as a representative result in Figs. 3 and 4 in main text, and there is no qualitative difference among the dimer-allowed circuits as shown in the following.

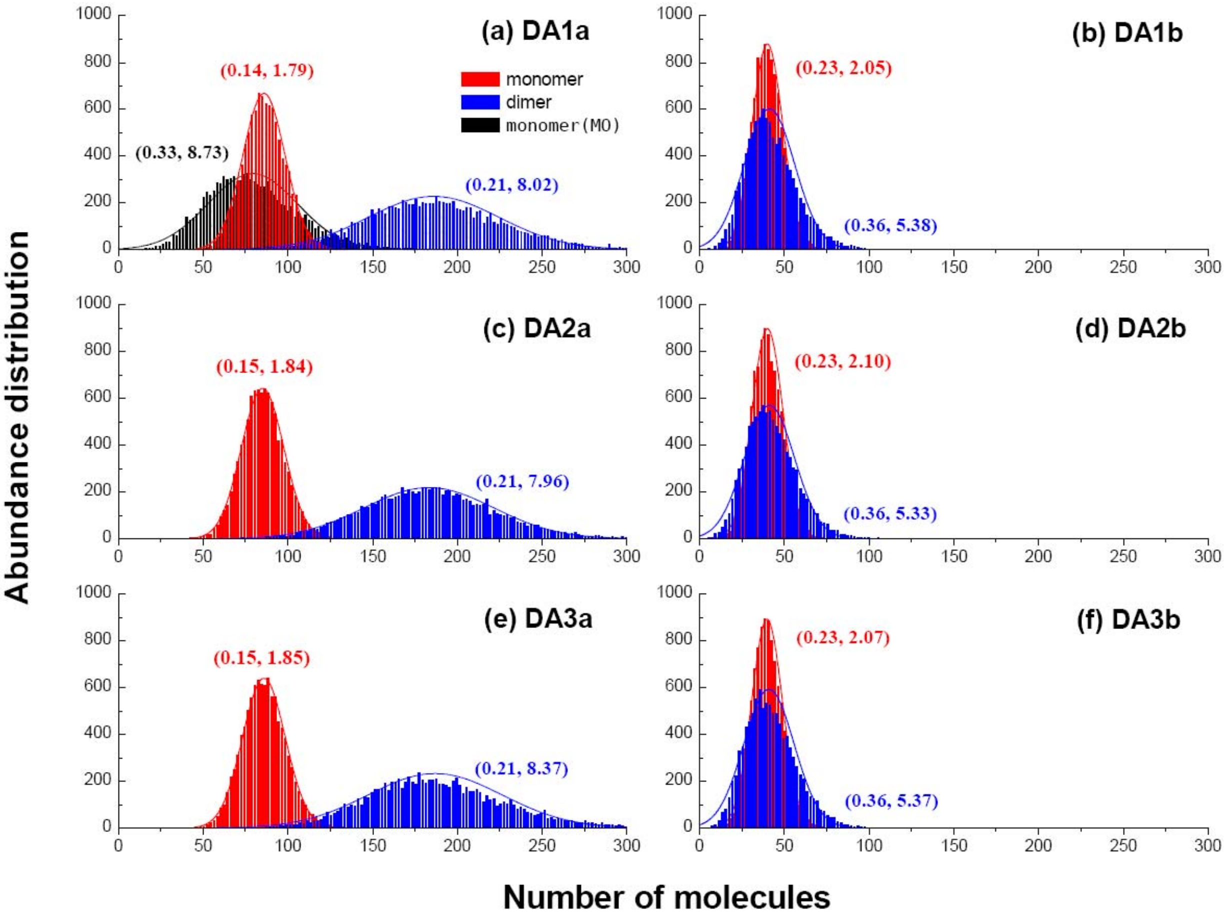

Figure S1. Abundance distribution of DA2 and DA3 circuits. DA1 is displayed for comparison. Left panels (a,c,e) shows the cases where the dimer half-life is 10 times longer than monomer half-life, while for the right panels (b,d,f), dimer half-life is twice the monomer-half-life. The two values in the parentheses denote coefficient of variation and Fano factor, respectively. Here the dissociation constant is fixed at 20 nM.

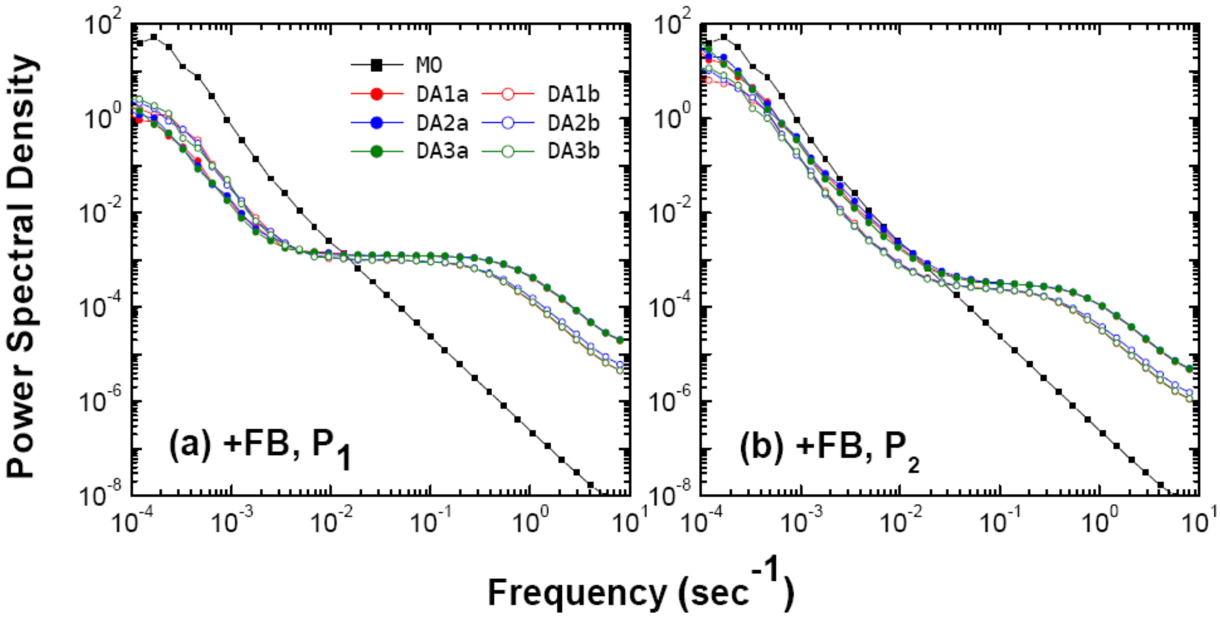

Figure S2. Power spectral density of monomer (a) and dimer (b) level fluctuation in positive autogenous circuits with distict topology (MO, DA_1, DA_2, and DA_3). K_1 is fixed at 20 nM. All the systems show the mid-frequency “whitening” as well as low-frequency reduction of noise regardless of the detailed regulatory pathways.

2. Effects of protein dimerization in negative autogenous circuit

Protein dimerization provides an independent control of noise. Genetic circuits with totally different topology or control logic may share the same effects as we have seen in positive autogenous circuits. Results for negative feedback circuits are shown in Fig. S2 (abundance distribution). In Fig. S3 we compares the modification of power spectra due to the presence of dimer state both in positive and negative feedback control.

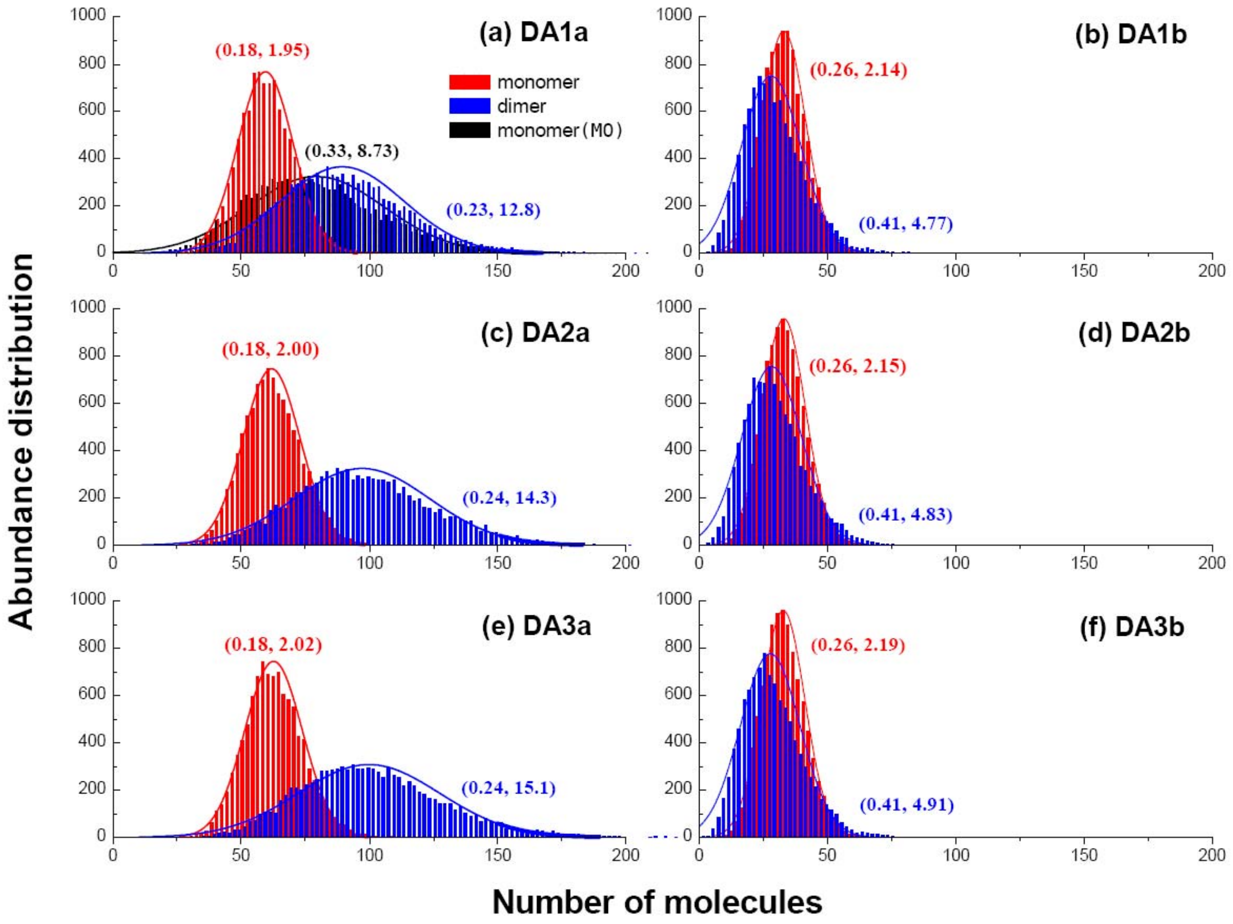

Figure S3. Abundance distribution of negative feedback circuits. Left panels (a,c,e) shows the cases where the dimer half-life is 10 times longer than monomer half-life, while for the right panels (b,d,f), dimer half-life is twice the monomer-half-life. The two values beside each histogram denote coefficient of variation and Fano factor, respectively. Here the dissociation constant is fixed at 20 nM.

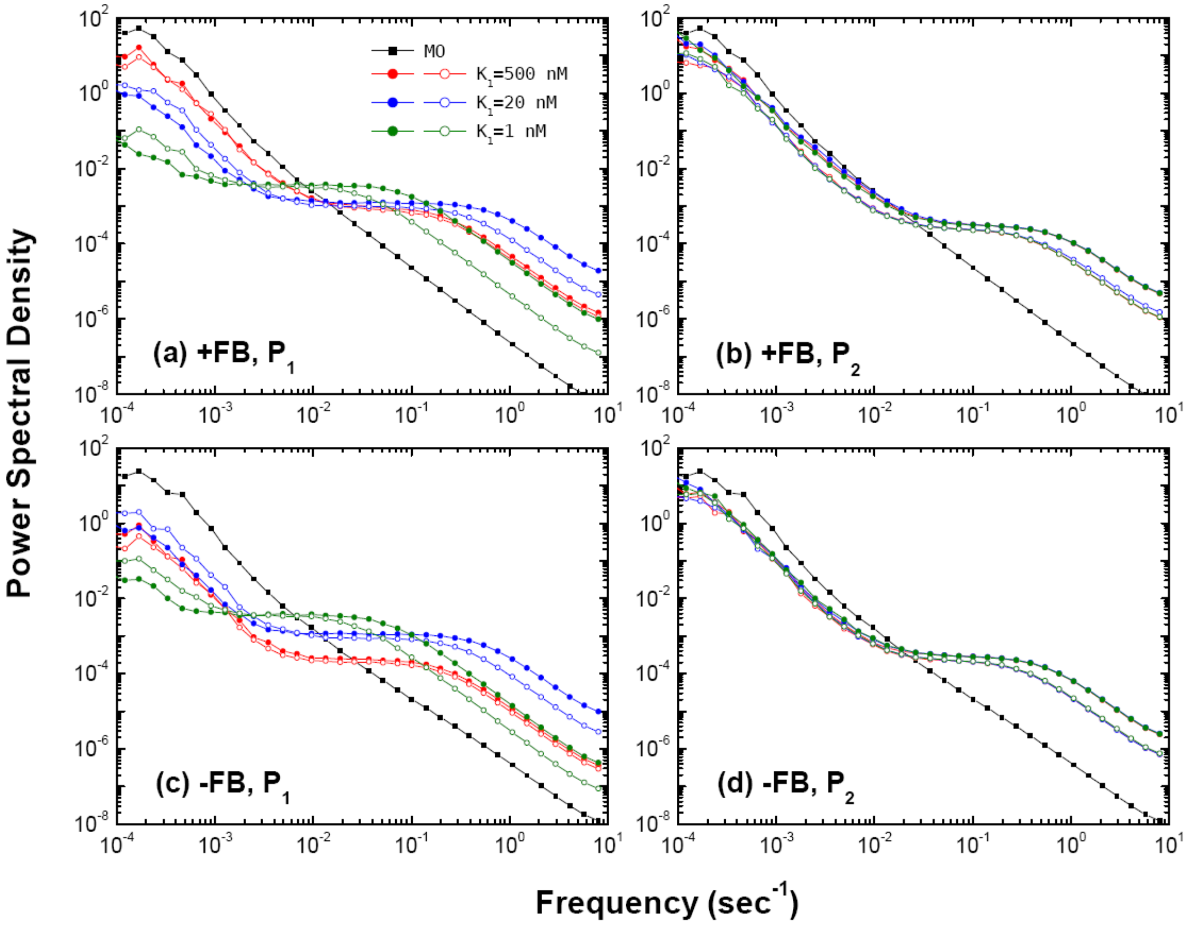

Figure S4. Power spectral density of positive (a,b) and negative (c,d) feedback circuits, where the circuit topology is fixed with DA_1. Filled (empty) circles denote the case where $\gamma_2 = 0.1\gamma_1$ ($\gamma_2 = \gamma_1/2$). As in the positive circuits, all the negative autogenous circuits also show the mid-frequency "whitening" as well as low-frequency reduction of noise regardless of the detailed regulatory pathways.

3. Power spectra of toggle switch

Toggle switch shows similar power spectra to those of autogenous circuits. Here are the cases where the active form of repressor protein is dimer. As the binding affinity gets higher (smaller K_1), overall noise tends to be reduced much like the autogenous circuits.

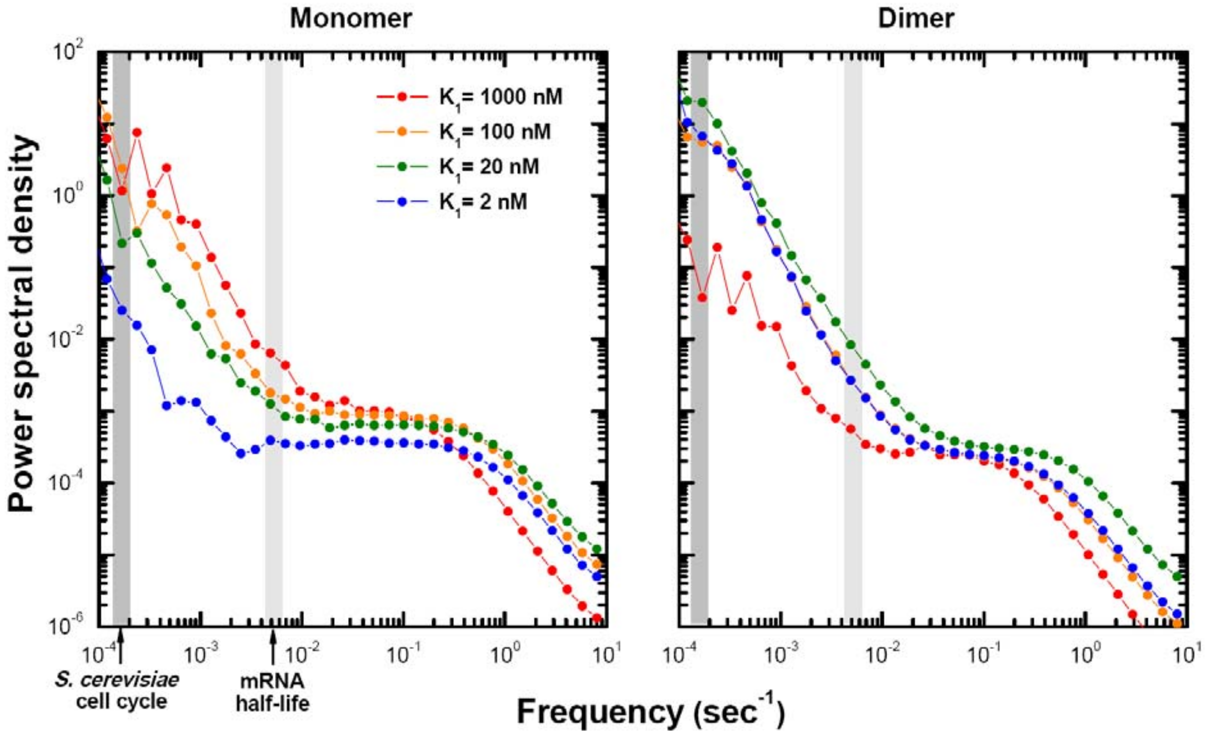

Figure S5. Power spectral density of toggle switch where the dimer is the active form of repressor and is formed exclusively in cytosol (similar to the autogenous circuit DA_1). Shown is the one species of protein, but the other species show similar characteristics due to the symmetry of the model system considered.
